# COVID-19 vaccination side effects among the child age group: a large cross-sectional online based survey in Saudi Arabia

**DOI:** 10.1186/s12879-022-07905-2

**Published:** 2022-12-06

**Authors:** Hassan Alwafi, Abdallah Y. Naser, Abdulelah M. Aldhahir, Ahmad Alhazmi, Areen Naif Alosaimi, Rasha Abdulaziz Mandili, Zaid Majeed, Emad Salawati, Rakan Ekram, Mohammed Samannodi, Hamza Assaggaf, Mohammed Almatrafi, Jaber S. Alqahtani, Safaa Mohammed Alsanosi, Faisal Minshawi

**Affiliations:** 1 Faculty of Medicine, Umm Al-Qura University, Mecca, Saudi Arabia; 2 Al-Noor Specialist Hospital, Mecca, Saudi Arabia; 3 Department of Applied Pharmaceutical Sciences and Clinical Pharmacy, Faculty of Pharmacy, Isra University, Amman, Jordan; 4 Respiratory Therapy Department, Faculty of Applied Medical Sciences, Jazan University, Jazan, Saudi Arabia; 5 Department of Family Medicine, Faculty of Medicine, King Abdulaziz University, Jeddah, Saudi Arabia; 6 School of Public Health and Health Informatics, Umm Al-Qura University, Mecca, Saudi Arabia; 7 Department of Laboratory Medicine, Faculty of Applied Medical Sciences, Umm Al-Qura University, Mecca, Saudi Arabia; 8 Department of Pediatrics, Faculty of Medicine, Umm Al-Qura University, Mecca, Saudi Arabia; 9 Department of Respiratory Care, Prince Sultan Military College of Health Sciences, Dammām, Saudi Arabia; 10 Department of Laboratory Medicine, Faculty of Applied Medical Sciences, Umm Al-Qura University, Mecca, Saudi Arabia

**Keywords:** COVID-19, Vaccination, Side effects, Children, Saudi Arabia

## Abstract

**Background:**

Multiple vaccines have been tested in clinical trials for their efficacy and safety. In Saudi Arabia, Pfizer–BioNTech or Moderna were approved for children, however, previous studies to report their safety profile are limited. This research aims to understand the side effect of children's vaccination against SARS-CoV-2 infection in Saudi Arabia.

**Methods:**

This was an observational retrospective cross-sectional study was conducted using an online survey in Saudi Arabia from March to May 2022. The inclusion criteria were parents aged 18 years and above who live in Saudi Arabia and have vaccinated their children. The self-reported questionnaire was adopted from published studies to investigate the study objectives Descriptive statistics were used to describe patients’ demographic characteristics, continuous data were reported as mean ± S.D., categorical data were reported as percentages (frequencies), and logistic regression was used to identify predictors of persistent post-COVID-19 symptoms.

**Results:**

This study had a total of 4,069 participants. Only 41.9% of the participants reported that their child(ren) had been infected with the coronavirus. 2.00 was the median number of children (IQR: 1.00–4.00). More than half of the study participants (64.2%) reported that a family member had been infected with the coronavirus. Both parents received COVID-19 vaccination, according to most participants (88.7%). Most participants (70.5%) stated that all children who met the vaccination criteria had received the vaccine. Most participants (83.5%) said their child or children had two doses of their vaccine, and about half (50.4%) of those who received the vaccine reported experiencing side effects. In addition, the majority (78.9%) reported that the side effects appeared within one day of receiving the vaccine, and nearly two-thirds (65.7%) reported that the side effects lasted between one and three. A total of 11,831 side effects cases were documented. Pain at the injection site, hyperthermia, and fatigue were the most reported side effects, accounting for 15.3%, 14.1%, and 13.2%, respectively.

**Conclusion:**

It appears that the side effects of the COVID-19 vaccine for children are minor, tolerable, and like those described previously in clinical trials. Our data should encourage the public about the safety of receiving the COVID-19 vaccine for children.

## Background

On 11 March 2020, the Coronavirus Disease 2019 (COVID-19) pandemic was declared [1]. Globally, communities have been impacted by the pandemic's rapid progress. The emerging Severe Acute Respiratory Syndrome Coronavirus-2 (SARS-CoV2) is the cause of the disease. It can be transmitted through direct contact or by respiratory droplets. In addition, it can impact the respiratory as well as gastrointestinal systems, causing symptoms ranging from the common cold, pneumonia, acute respiratory distress syndrome (ARDS), to multi-organ failure, and death [2, 3]. According to the World Health Organization (WHO), as of May 27, 2022, COVID-19 has a cumulative total of 525,467,084 confirmed cases and 6,285,171 deaths [1]. In Saudi Arabia, the COVID-19 has spread and affected a total of 518,143 confirmed cases, and a reported overall case-fatality rate of 1.57% [4]. Attempts are being made to slow the spread of COVID-19 due the high transmission rate of COVID-19 [5]. For a long time, there have been multiple strategies to mitigate the spread of infectious diseases, and the most effective strategy is to isolate confirmed cases [5, 6]. Globally, lockdowns have been imposed with other strict restrictions, including curfews, using face masks and hand hygiene to ensure social distancing as well as suppress the spreading of infection [5, 7].

Since COVID-19 is declared as a pandemic, vaccination brought hope to control this condition. There are different COVID-19 vaccines approved by WHO, such as The Pfizer/BioNTech vaccine and AstraZeneca/AZD1222 vaccines [7, 8]. Vaccinations can provide excellent protection from serious illness, hospitalization, and death. Generally, they appeared to be safe and effective especially in double doses [9, 10]. Thirty vaccines went for clinical trials in the advanced stage of vaccine development. In early December 2020, Pfizer–BioNTech received a temporary emergency use authorization, and at the end of December, it received marketing authorizations for active immunization to prevent SARS-CoV-2 infection [11, 12]. In Saudi Arabia, Pfizer–BioNTech and Moderna vaccines are approved and currently available for adults and children > 4 years old [13].

This vaccination underwent a safety evaluation to observe any adverse reaction following the injection of either dose for adults. In most clinical trials, injection-site pain was the most frequently reported local adverse reaction. In addition, moderate to mild fever, headache, and fatigue were frequently reported as adverse systemic reactions. A relatively small number of patients experienced a severe systemic reaction [14–16]. Even though numerous trials on the safety and efficacy of vaccinations have been conducted, these trials were not conducted for a longer duration after vaccination and did not include all age groups. Recently, FDA approved the use of Moderna and Pfizer–BioNTech COVID-19 vaccines for Children 5–17 years of Age [17]. Previous studies in Saudi Arabia focused on different clinical and psychological perspectives that explored the impact of COVID-19 on the health status of Saudi patients and population [10, 18–27]. However, there are no large scale studies that have explored safety profile of COVID-19 vaccination among children in Saudi Arabia. Consequently, there is a need for data regarding the COVID vaccine in Saudi Arabia, particularly in terms of safety and efficacy evaluation, and this study can serve as a steppingstone for further analytic research. This study aimed to describe the most common side effects of the approved COVID-19 vaccines in Saudi Arabia among children aged 5 to 17 years.

## Methods

### Study design

This was an observational retrospective cross-sectional study using an online survey conducted in Saudi Arabia between March and May 2022. Parents aged 18 years and above and living in Saudi Arabia were eligible to complete the survey.

### Study population and sampling strategy

This study used a convenience sample to recruit the study population. The study was conducted among the general population of Saudi Arabia, including all the geographic regions of Saudi Arabia (Western, Eastern, Central, Northern, and Southern) from March to May 2022, using an online questionnaire. The questionnaire was formulated in Arabic and distributed through social media platforms such as WhatsApp, Twitter, and Snapchat). The study sample was invited using a survey link. The inclusion criteria were parents aged 18 years and above who live in Saudi Arabia (Saudis and non-Saudis) and have vaccinated their children. The survey link was re-posted once weekly to increase the response and make it reachable to the general population. The cover letter clearly stated the study's aims and objectives.

The questionnaire was originally prepared in English. The original questionnaire was translated into Arabic utilizing the forward and backward translation technique. Two professional clinicians and academics assessed the Arabic version of the study and affirmed that participants would have no trouble understanding it. The Arabic version of the questionnaire was then administered to 30 participants in Saudi Arabia who met the inclusion criteria for the study. Participants were asked about the clarity and readability of the questionnaire, as well as whether any questions were difficult to understand. Participants were also asked whether any of the questions were offensive or unpleasant. Participants reported that the questionnaire was straightforward to comprehend and complete.

### Study tool

The self-reported questionnaire was adopted from published studies to investigate the study objectives [28–30]. After structuring the questionnaire using a Google Survey, data were collected for three months, from March to May 2022. Data were kept safe with authorized access only. The first section comprised of seven-items that described the demographic characteristics of the study participants. The second section comprised of three-items and asked the participants about number of their children and their disease history. The third section comprised of six-items and explored COVID-19 infection and allergy profile of the study participants. The fourth section comprised of eight-items and explored COVID-19 vaccination profile of the study participants.

### Sample size estimation

The target sample size was determined in accordance with WHO recommendations for the minimum sample size required for a prevalence study [31]. The sample size required was 385 participants based on a 95% confidence interval, a standard deviation of 0.5, and a 5% margin of error.

### Statistical analysis

Descriptive statistics were used to describe patients’ demographic characteristics, continuous data were reported as mean ± S.D., categorical data were reported as percentages (frequencies), and logistic regression was used to identify predictors of persistent post-COVID-19 symptoms. For the logistic regression, the independent variables were the presence of allergy, chronic conditions, or the smoking status of the parents and the dependent variable was defined as patient who had persistent post-COVID-19 symptoms for more than 4 weeks.

A two-sided p < 0.05 was considered statistically significant. The statistical analyses were carried out using S.P.S.S. (version 27).

## Results

### Sociodemographic characteristics of the study participants:

This study had a total of 4,069 participants. The majority of them (92.8%) were Saudis. Around half of participants (52.7%) reported living in the western region. Around half of the parents who took part had a bachelor's degree (fathers 48.4% and mothers 50.4%). The average monthly income of one-third of the research participants (35.4%) was between 10,000 and 20,000 SAR, Table 1.

**Table 1:** Sociodemographic characteristics of the study participants

Demographic variable	Frequency	Percentage (%)
Nationality		
Saudi	3776	92.8
Area of residency		
Central area	598	14.7
Eastern area	403	9.9
Western area	2144	52.7
Northern area	468	11.5
Southern area	456	11.2
Father’s educational level		
Intermediate degree or lower	618	15.2
Secondary education	964	23.7
Bachelor’s degree	1969	48.4
Higher degree	517	12.7
Mother’s educational level		
Intermediate education or lower	802	19.7
Secondary education	863	21.2
Bachelor’s degree	2051	50.4
Higher education	354	8.7
Family’s monthly income		
Less than 5000 SAR	623	15.3
5000–10,000 SAR	1172	28.8
10,000–20,000 SAR	1440	35.4
More than 20,000 SAR	834	20.5
Does any of the parents’ smoke?		
Father smoker	1021	25.1
Mother smoker	61	1.5
Both of them are smokers	138	3.4
None of the parents is smoker	2848	70.0

*SAR* Saudi Arabian Riyal

When parents were asked how many children they had, the median answer was three (IQR: 2.00–5.00). Around 14.7% of the participants said their children have chronic conditions, the most common of which were asthma and type 1 diabetes mellitus (T1DM), with 5.3% and 2.3%, respectively, Table 2.

**Table 2:** Number of children and their disease history

Variable	Frequency	Percentage (%)
How many children do you have?		
Median number of children (IQR)	3.00 (2.00–5.00)	
Does any of your children suffer from chronic diseases?		
Yes	598	14.7%
If yes, what are these diseases? (n = *598)*		
Asthma	32	5.3%
Type 1 diabetes mellitus	14	2.3%
Blood disease (ex: anaemia)	13	2.2%
GIT system diseases	5	0.9%
Thyroid gland diseases	5	0.8%
CNS system diseases	4	0.7%
Cardiovascular diseases	4	0.6%
Tumors	2	0.4%
Others	16	2.7%

### SARS-CoV-2 infection and vaccination side effect profile

Only 41.9% of the participants reported that their child(ren) had been infected with the coronavirus. 2.00 was the median number of children (IQR: 1.00–4.00). Most children (89.2%) had been infected with the disease for at least six months.

At least one of their children has an allergy, according to one-quarter of the survey participants (25.0%). Most children with allergies (78.2%) were allergic to food. Around half of them (48.3%) said their allergic child(ren) had an allergic reaction in the previous month, Table 3.

**Table 3:** COVID-19 infection and allergy profile

Variable	Frequency	Percentage (%)
Have any of your children contracted corona virus disease?		
Yes	1705	41.9
If yes, how many of them were infected?		
Median number of children (IQR)	2.00 (1.00–4.00)	
When did your children contracted corona virus diseases? (n = 1705)		
During the past week	31	1.8
During the past 2 weeks	44	2.6
During the past 4 weeks	109	6.4%
During the past 6 months	806	47.3%
During the past year	714	41.9%
Do any of your children has allergy of any type?		
Yes	1017	25.0%
If yes, what is the type of the allergy? (n = 1017)		
Food allergy	795	78.2%
Medication-related allergy	222	21.8%
If your child(ren) has an allergy, when was the last time it was exposed? (n = 1017)		
During the past day	87	8.6%
During the past 2 days	60	5.9%
During the past week	111	10.9%
During the past 2 weeks	93	9.1%
During the past month	140	13.8%
During the past 3 months	129	12.7%
During the past year	134	13.2%
More than 1 year ago	261	25.7%

More than half of the study participants (64.2%) reported that a family member had been infected with the coronavirus. Both parents received COVID-19 vaccination, according to most participants (88.7%). Most participants (70.5%) stated that all children who met the vaccination criteria had received the vaccine. Most participants (83.5%) said their child or children had two doses of their vaccine, and about half (50.4%) of those who received the vaccine reported experiencing side effects. In addition, the majority (78.9%) reported that the side effects appeared within one day of receiving the vaccine, and nearly two-thirds (65.7%) reported that the side effects lasted between one and three days, Table 4.

**Table 4:** COVID-19 vaccination profile

Variable	Frequency	Percentage (%)
Has anyone else in the family had corona virus diseases?		
Yes	2612	64.2
Has any other family member received the COVID-19 vaccine?		
The father only	142	3.5
The mother only	208	5.1
Both	3609	88.7
None	110	2.7
Have all of your children (who are and are allowed to receive the vaccine) received the COVID-19 vaccine?		
No, none of them received the vaccine even though they were allowed to take it	696	17.1
Yes, all those who meet the conditions receive the vaccine	2869	70.5
Yes, but some of them did not receive the vaccine despite the application of the necessary conditions for taking the vaccine	505	12.4
If your answer was yes for the previous question, how many doses of the COVID-91 vaccine did your child(ren) have? (n = *3373)*		
One dose	557	16.5
Two doses	2816	83.5
Did your child(ren) have any side effects after getting the vaccine? (n = 3373)		
No, none of them suffered from side effects	1673	49.6
Yes, some of them experienced side effects	1235	36.6
Yes, they all experienced side effects	465	13.8
Do your children have the same side effects? (n = 1700)		
No, it was different from one child to another	804	47.3
Yes, they all had the same symptoms	896	52.7
When did your child/children’s symptoms start after getting the vaccine? (n = 1700)		
After 1 day	1341	78.9
After 2 days	260	15.3
After more than 3 days	97	5.7
What is the average duration of side effects in your children? (n = 1700)		
1–3 days	1117	65.7
3–5 days	337	19.8
5–7 days	141	8.3
1–4 weeks	39	2.3
More than 1 month	68	4.0

**Fig. 1:**
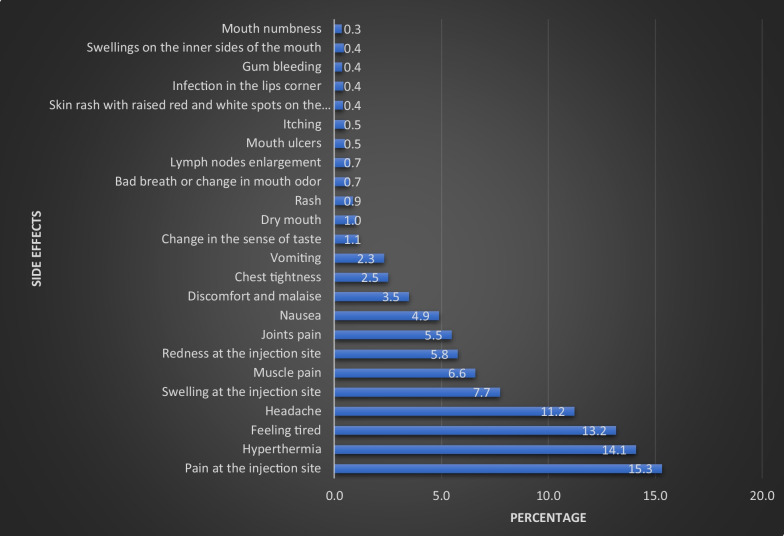
Distribution of reported side effects related to COVID-19 vaccine

When we requested parents to report side effects encountered by their children, a total of 11,831 cases were documented. Pain at the injection site, hyperthermia, and feeling tired were the most reported side effects, accounting for 15.3%, 14.1%, and 13.2%, respectively, Fig. 1.

Binary logistic regression analysis showed that smoking status of the parents, having allergy and having other comorbidities were risk factors of having persistent post-COVID-19 symptoms (p ≤ 0.05), Table 5.

**Table 5:** Binary logistic regression analysis

Variable	Odds ratio (95% confidence interval)	p-value
Does any of the parents’ smoke?		
No (Reference group)	1.00	
Yes	1.76 (1.01–3.08)	0.046
Does any of your children suffer from chronic diseases?		
No (Reference group)	1.00	
Yes	2.62 (1.44–4.76)	0.002
Do any of your children has allergy of any type?		
No (Reference group)	1.00	
Yes	2.23 (1.28–3.8)	0.005

## Discussion

The present study showed that most parents and their children received COVID-19 vaccine, and COVID-19 vaccine hesitancy was low. Moore et al. found a low rate of vaccination hesitancy among Brazilians [32]. COVID-19 vaccination hesitancy is widespread, ranging from 2.8% in Brazil to 35.2% in Qatar [32, 33]. Furthermore, Altulaihi et al. found that 27% of respondents in Saudi Arabia were hesitant to receive the vaccine [34]. Our previous study showed that most parents were welling to vaccinate their children against COVID-19 vaccine, and COVID-19 vaccine hesitancy was low [10]. Moreover, according to Temsah et al. 47.6% of 3,167 Saudi Arabian parents have decided to vaccinate their children against COVID-19. Inadequate safety information in children and concerns about side effects were the most common reasons for the refusal [10]. Common factors causing hesitation include ambiguity about the need for immunization and uncertainties about vaccine safety and efficacy [35]. Sociodemographic factors related with parental vaccine reluctance vary by place and circumstance [36]. Cognitive biases, personal beliefs, and vaccination as a social contract or norm are highlighted in studies about the psychology of hesitancy and how parents respond to interventions [37]. Presumptive or announced approaches to vaccine recommendations, motivational interviewing, and the use of immunization delivery strategies such as standing orders and reminder/recall programs are evidence-based ways for addressing vaccine hesitancy. Increasing school vaccination requirements can increase vaccination rates, but policy decisions must take local context into account [37].

In our study, more than half of the people reported that their children had adverse effects from their vaccination and that the side effects lasted one to three days. The Centers for Disease Control and Prevention reported that children and teenagers may experience some adverse effects after receiving the COVID-19 vaccination, which may interfere with their ability to do daily activities, but that these side effects should subside within a few days [38]. According to the Saudi Ministry of Health, most vaccine adverse effects are mild to moderate, develop within three days of vaccination, and subside within one to two days [39]. Similar to other vaccines, COVID-19 vaccinations can have side effects, however, the majority of which are minor or moderate and fade away on their own within a few days, according to the World Health Organization [40].

The most reported side effects in our study were pain at the injection site, fever, and tiredness. A systematic review of the safety, immunogenicity, and efficacy of COVID-19 vaccines in children and adolescents showed that COVID-19 vaccines had good safety profiles in children and adolescents and that injection site pain, fatigue, headache, and chest pain were the most common adverse events [41]. According to the Centres for Disease Control and Prevention, the most common side effects observed after receiving the COVID-19 vaccination are mild headaches, pain in the arm where the shot was administered, and tiredness [38]. Furthermore, Alamer et al. conducted a study in Saudi Arabia on the side effects of the COVID-19 Pfizer–BioNTech mRNA Vaccine in children aged between 12 and 18 years old and found that 90% of the children reported redness or pain at the injection site, 67% fatigue, 59% fever, 55% headache, 21% nausea or vomiting, and 20% chest pain and shortness of breath, with only 2% reporting joint or bone pain [28].

According to the CDC's Vaccine Adverse Event Reporting System, more than 90% of post-vaccination adverse event reports among children and young people were not for significant symptoms and included dizziness, fainting, nausea, headache, and fever [38]. Centers for Disease Control and Prevention declared several severe adverse events that could occur after the COVID-19 vaccination, such as anaphylaxis, myocarditis, pericarditis, and Guillain-Barré syndrome, but these are rare [38].

In our study, most vaccine side effects (~ 80%) tend to occur on the first day of vaccination and resolve within 1–2 days. In contrast, long-lasting side effects were noticed in minimal participants of our population (~ 4%). Compared to our study, Kaur R et al. have documented in their systematic review that most COVID-19 vaccine side effects are acute and usually resolved in 3–4 days [42]. Additional studies have reported similar findings of vaccine’s side effects duration like our study [29, 43].

The confidence and trust of the public in vaccines and medications are usually built based on high quality research, ethical, scientific, and professional standards [44]. The ability of scientist and health care providers to provide answers based on scientific evidence are needed to help guide and encourage the public to follow new policies and interventions [44].The result of this study shows that the majority of the side effects are minor and tolerable, which should encourage the public about the safety of receiving the COVID-19 vaccine for children.

There are certain limitations to our research. The first limitation is that because the present study included a self-administered survey, recall bias may affect the replies of the participants. The second limitation is that the participants were not limited to one response per person, which could lead to an overestimation or under-estimation of the presence of side effects. The third limitation is that the study's findings were based on survey data, which means that, like any other cross-sectional study, the results cannot be used to infer causality.

## Conclusion

This research contributes to understanding the side effect of children vaccination against SARS-CoV-2 infection in Saudi Arabia. In this report, the most prevalent side effects were pain at the injection site, hyperthermia, and tiredness. These side effects are minor, tolerable, and like those described in clinical trials, demonstrating that COVID-19 vaccinations have safe profiles. Further studies with larger populations are necessary to evaluate the safety of COVID-19 vaccinations.

## Data Availability

All data are available on reasonable request from the corresponding author.

## Abbreviations

COVID-19: Coronavirus Disease 2019
SARS-CoV2: Severe Acute Respiratory Syndrome Coronavirus-2
WHO: World Health Organization
T1DM: Type 1 diabetes mellitus
