# Correction: COVID-19 vaccination side effects among the child age group: a large cross-sectional online based survey in Saudi Arabia

**DOI:** 10.1186/s12879-024-09950-5

**Published:** 2024-10-10

**Authors:** Hassan Alwaf, Abdallah Y. Naser, Abdulelah M. Aldhahir, Ahmad Alhazmi, Areen Naif Alosaimi, Rasha Abdulaziz Mandili, Zaid Majeed, Emad Salawati, Rakan Ekram, Mohammed Samannodi, Hamza Assaggaf, Mohammed Almatraf, Jaber S. Alqahtani, Safaa Mohammed Alsanosi, Faisal Minshawi

**Affiliations:** 1 Department of Pharmacology and Toxicology, College of Medicine, Umm Al-Qura University, Mecca, Saudi Arabia; 2 Al-Noor Specialist Hospital, Mecca, Saudi Arabia; 3 Department of Applied Pharmaceutical Sciences and Clinical Pharmacy, Faculty of Pharmacy, Isra University, Amman, Jordan; 4 Respiratory Therapy Department, Faculty of Applied Medical Sciences, Jazan University, Jazan, Saudi Arabia; 5 Department of Family Medicine, Faculty of Medicine, King Abdulaziz University, Jeddah, Saudi Arabia; 6 School of Public Health and Health Informatics, Umm Al-Qura University, Mecca, Saudi Arabia; 7 Department of Laboratory Medicine, Faculty of Applied Medical Sciences, Umm Al-Qura University, Mecca, Saudi Arabia; 8 Department of Pediatrics, Faculty of Medicine, Umm Al-Qura University, Mecca, Saudi Arabia; 9 Department of Respiratory Care, Prince Sultan Military College of Health Sciences, Dammām, Saudi Arabia; 10 Department of Laboratory Medicine, Faculty of Applied Medical Sciences, Umm Al-Qura University, Mecca, Saudi Arabia

Following publication of the original article [1], we have been notified that the first affiliation was missing the department name.

Originally published affiliation no. 1: Faculty of Medicine, Umm Al-Qura University, Mecca, Saudi Arabia

Correct affiliation: Department of Pharmacology and Toxicology, College of Medicine, Umm Al-Qura University, Mecca, Saudi Arabia

The original article has been corrected.

## References

[1] Alwafi, et al. BMC Infect Dis. 2022;22:911. 10.1186/s12879-022-07905-2. 10.1186/s12879-022-07905-2 PMC9724422 36474174

